# Estimating equivalence scales and non-food needs in Egypt: Parametric and semiparametric regression modeling

**DOI:** 10.1371/journal.pone.0256017

**Published:** 2021-08-20

**Authors:** Fuad A. Awwad, Suzan Abdel-Rahman, Mohamed R. Abonazel

**Affiliations:** 1 Department of Quantitative Analysis, College of Business Administration, King Saud University, Riyadh, Saudi Arabia; 2 Department of Demography and Biostatistics, Faculty of Graduate Studies for Statistical Research, Cairo University, Giza, Egypt; 3 Department of Applied Statistics and Econometrics, Faculty of Graduate Studies for Statistical Research, Cairo University, Giza, Egypt; Institute for Economic Forecasting, Romanian Academy, ROMANIA

## Abstract

This paper investigated the appropriate specifications of Engel curves for non-food expenditure categories and estimated the deprivation indices of non-food needs in rural areas using a semi parametric examination of the presence of saturation points. The study used the extended partial linear model (EPLM) and adopted two estimation methods—the double residual estimator and differencing estimator—to obtain flexible shapes across different expenditure categories and estimate equivalence scales. We drew on data of the Egyptian Household Income, Expenditure, and Consumption Survey (HIEC). Our paper provides empirical evidence that the rankings of most non-food expenditure categories is of rank three at most. Rural households showed high economies of scale in non-food consumption, with child’s needs accounting for only 10% of adult’s non-food needs. Based on semi-parametrically estimated consumption behavior, the tendency of non-food expenditure categories to saturate did not emerge. While based on parametrically estimated consumption behavior, rural areas exhibited higher deprivation indices in terms of health and education expenditure categories, which indicates the need to design specific programs economically targeting such vulnerable households.

## Introduction

The Engel curve is the heart of empirical demand analysis. It depicts the relationship between total expenditures and a particular good or service. Determining the correct specification of an Engel curve has important implications for consumption patterns. It approximates household welfare by illustrating the gradient in welfare levels in response to changes in expenditure shares and, most importantly, estimating demographic impact on demand for essential items, and identifying equivalence scales. It also provides a useful instrument for designing tax policies and assessing their impact on household welfare, which helps policymakers grant an acceptable level of welfare to low-income households [1–3]. Investigating the appropriate specification of the Engel curve has been a topic of great interest over the past few decades. A considerable body of empirical literature exists for the developed countries. However, such studies are scarce in developing countries, including Egypt. Recently, empirical studies have been more interested in another implication; namely, employing a saturating Engel curve for poverty measurement [4–7], which considers our focal objective.

The Central Agency for Public Mobilization and Statistics (CAPMAS) announced that poverty rates in Egypt continue to rise. The overall poverty rate has increased constantly since 2004/05; from 19.6% in 2004/05 to 25.2% in 2011, continually rising until it reached 27.8% in 2015. Rural areas witnessed the highest poverty rates, about 40% of rural households live below the poverty line. Although poverty constitutes a substantial challenge for socioeconomic development and diminishes household well-being, efforts to measure its level and structure have been limited.

The poverty line is a very argumentative and subjective concept. It determines in advance the basic need to define the poor, although household preferences control the prevailing consumption pattern and realistically constitute the essential commodities for any society. Consumption deprivation identifies the poor without determining a prior poverty line or using subjective norms [4, 5, 8]. Deprivation is a broader concept of poverty, it measures the threshold required to satisfy the basic needs of life for all, not just for the extremely poor. Deprivation is the main driver of an increase in expenditures on a specific commodity, as income increases up to a critical threshold (i.e., the saturation level) at which consumption needs are satisfied. Saturation refers to the case in which expenditure on a particular commodity will eventually cease to increase in response to any further incremental increase in income; or the point at which income elasticity reaches zero. The saturation point is considered as a deprivation point and can be used to measure consumption deprivation indices of non-food basic needs. The saturation level of commodity-specific consumption is considered a norm set by the community to determine the deprivation threshold. In this context, the paper aims to estimate saturation points for non-food categories to measure the deprivation indices for rural households in Egypt.

It is difficult to establish a consensus on a poverty measure in terms of its level or its structure. Nevertheless, we embrace the idea that investigating consumption patterns will provide a new pathway for avoiding dubious and outdated poverty methods. The deprivation index is free of any subjective selection bias and is also variable over time as it reflects the prevailing consumption patterns in society. Moreover, measuring consumption deprivation can greatly improve vulnerable groups’ welfare in specific expenditure categories.

How robust the existence of the saturation point is depends on the Engel curve’s shape [6, 9]. After reviewing the Engel curve literature paying attention to specification issues, we found that most studies explored the Engel curve’s specification for the food expenditure group, while the non-food expenditure categories have not received the same level of attention [10]. Many studies in developed countries have posited that the Engel curve for food is linear, while it is more likely to exhibit non-linearity across other non-food needs [1, 2]. The empirical studies in developing countries have supported a quadratic shape for the food Engel curve [10–13]. These findings should be taken with caution, as Engel curve specifications respond to the consumption pattern of each country. In addition, it is controlled by several factors such as total expenditure endogeneity, outlier problems, and shape invariance features across demographic types. Despite these methodological issues and the substantial implications of the Engel curve, no attempt has been conducted to examine its correct specifications in Egypt based on its specific consumption patterns. Therefore, one of our study’s aims is to fill this gap.

Some parametric specifications have imposed a saturating Engel curve as a Sigmoid function [14] and a hyperbolic function [4, 8], in which the curve possesses a saturation point. Advanced non-parametric and semi-parametric techniques substantially increase the ability to discover empirical regularities and provide a broader scope for investigating the Engel curve’s economic features. The non-parametric approach proposes an attractive alternative compared with parametric techniques by enabling the data to determine the conditional mean relationship and the existence of a saturation point without the *a priori* assumptions implied in the parametric approach. Moneta and Chai [6] proposed non-parametric measurements for weak and strong saturation states by examining changes in confidence bands across total expenditure levels [6].

Despite the flexibility of the non-parametric technique, it faces major drawbacks. It suffers from a slow convergence rate and imprecise estimates in finite samples due to the curse of dimensionality (which arises in highly dimensional data and with a limited number of observations). The curse of dimensionality restricts the estimation of Engel relationships with an accepted degree of precision as the data requirements increase dramatically as the number of non-parametric variables increase. Therefore, empirical studies that have applied non-parametric techniques have been restricted to using only one independent variable (i.e., total expenditure) and did not account for the heterogeneity of households and the fact that there are several factors other than total household expenditure that significantly affect spending on commodities (e.g., household size, sex and age composition of the household, and the proportion of the elderly and adults) [6, 15]. In contrast to parametric and non-parametric techniques, the semi-parametric technique introduces a superior alternative by compromising between the parametric and non-parametric techniques [16, 17]. It allows total household expenditures to enter the model non-parametrically while other covariates are modeled parametrically. Accordingly, we used the semi-parametric regression technique to separate the effect of household composition and other observed factors and investigate the existence of saturation points without imposing restrictive assumptions.

Nominal expenditures are no longer an appropriate indicator of the standard of living as households are heterogeneous in size and composition. Deaton and Muellbauer [3] stated that equivalized expenditures yield more accurate estimates of household welfare and avoid overestimating the expenditure of large households [3]. The equivalence scale converts the nominal incomes of households with different demographic compositions to comparable measures. As we strive to obtain consistent estimates of an equivalence scale while allowing flexible shapes for Engel curve relationships across the various expenditure categories, we identify the equivalence scale semi-parametrically using the extended partial linear model (EPLM), which assumes base independence [2]. Moreover, we handled the measurement error and simultaneity in the total expenditure variable in a semi-parametric context.

This paper provides two innovations for measuring poverty in Egypt, one substantive and the other methodological. The substantive focus of this paper is measuring the non-food poverty rate using a broader concept of poverty “deprivation indices,” while the methodological aspect is concerned with investigating the correct specifications of the Engel curves for the non-food expenditure categories, estimating the equivalence scale semi-parametrically across all non-food expenditure categories to simultaneously provide comparable results across different households and various expenditure items, and finally estimating the saturation points for the non-food categories to establish deprivation indices for Egyptian households.

To achieve our objectives, the study is constructed in five sections. Following the introduction, Section 2 discusses the data sources and details the extended partial linear model, the estimation methods, and the procedures used to derive the saturation points. Section 3 analyzes household expenditure patterns in rural areas and provides estimates of the equivalence scales as well as parametric and semi-parametric regressions for non-food expenditure categories. Sections 4 discusses our findings and Section 5 concludes.

## Methods and materials

### Data sources

We utilize cross-sectional data from the Egyptian Household Income, Expenditure, and Consumption (HIEC) Survey carried out by the CAPMAS in 2015. Households surveyed in the HIEC are selected using a two-stage stratified random sampling approach. The data does not suffer from the problems of zero expenditure or “impossible” expenditure values (i.e., values that are too high or too low to be plausible) as they have been cleaned by the data team consisting of qualified statisticians from the Economic Research Forum [18].

There are substantial variations in the living standards between urban and rural areas that are due to the different economic, social, and demographic characteristics. This is clearly reflected in consumption patterns and inevitably in the specifications of the Engel curves. We are convinced that measuring deprivation for a homogenous population that shares the same set of preferences, tastes, and consumption behavior is more reliable. Fortunately, a large sample size enables us to analyze urban and rural areas separately, which increases analytical precision and further convenience. As we are interested in measuring deprivation indices in rural areas paying more attention to vulnerable households, our analysis is conducted using a rural data set consisting of 6,670 households. Ensuring that there is a sufficient number for each household composition left 6,191, dropping only 479 households (see Table 1). We control the sample design by giving different weights for the surveyed households. We retrieve our results based on sample weights to reflect the actual distribution and characteristics of the population. We used survey packages in the R program.

**Table 1:** Composition of rural households.

Number of Adults		Number of Children					
		0	1	2	3	4	Total
**1**	**Count**	357	30	51	54	28	520
	**%**	68.65	5.77	9.81	10.38	5.38	100
**2**	**Count**	731	238	610	717	269	2565
	**%**	28.5	9.28	23.78	27.95	10.49	100
**3**	**Count**	541	226	294	228	88	1377
	**%**	39.29	16.41	21.35	16.56	6.39	100
**4**	**Count**	436	288	201	121	44	1090
	**%**	40.00	26.42	18.44	11.10	4.04	100
**5**	**Count**	259	168	126	63	23	639
	**%**	40.53	26.29	19.72	9.86	3.60	100
**Total**	**Count**	2324	950	1282	1183	452	6191
	**%**	37.54	15.34	20.71	19.11	7.30	100

The HIEC includes modules on various aspects of household demographics and socioeconomic characteristics such as education, health, employment, and ownership of durables. CAPMAS used the latest classifications developed by the United Nations Statistics Division to classify and analyze the individual consumption expenditures incurred by households [19]. The HIEC includes a wide range of expenditure items (see S1 Table in S1 File).

Our paper specifically addresses the non-food expenditure categories to detect the appropriate specifications consistent with the observed expenditure patterns in rural areas. It is accurate to separate the food commodity group from the non-food commodity group; each has a different equivalent scale. Non-food goods are largely consumed by household members at the same time, unlike food goods, which are rivals for consumption. We consider six exhaustive non-food categories: clothing, housing, education, health, transport, and others. Table 2 reports the summary statistics for these expenditure categories.

**Table 2:** Summary statistics for non-food expenditure shares.

	Mean	Median	SD	Min	Max
Clothing	0.06	0.05	0.03	0.00	0.23
Housing	0.16	0.15	0.06	0.03	0.70
Education	0.04	0.02	0.04	0.00	0.62
Health	0.08	0.06	0.08	0.00	0.82
Transport	0.04	0.03	0.04	0.00	0.71
Other	0.20	0.19	0.07	0.03	0.75

Many reasons drive us to conduct analysis at the household level, such as the issue of intra-household distribution of income wherein resources are shared among household members. Moreover, some consumption items are practically measured at the household level, which enhances using the household as a reference point. We use total expenditures as an efficient indicator of the standard of living rather than income, as it more accurately estimates the permanent incomes of households. Income is very volatile and may deviate from the life cycle due to transient fluctuations. It is also difficult to measure income compared to consumption, especially in poor households in which the measurement error is severe [11, 20]. We distinguish poor households from better-off households based on patterns in total expenditures, a commonly accepted measure. We grouped households into different classes according to expenditure quintiles by dividing the ordered total expenditure variable into five equal-sized groups.

### Model

Suppose there are two households *A* and B, and consider B to be the reference household. Both households experience the same prices where *p*, the price, is a vector and *u* is the utility level. So,

$$E^{A}\left(p,u\right)=\frac{E^{B}\left(p,u\right)}{\Delta^{B}\left(p,u\right)},$$ (1)

where *E*^*i*^(*p*, *u*) is the expenditure function that gives a specific household *i* ∈ {*A*, *B*} utility *u* at the prevailing price *p*. The equivalence scale, Δ^*B*^(*p*, *u*), scales the expenditure of household *B* such that both households *A* and *B* have the same utility level, “Equivalent Expenditure.”

The base-independence assumption implies that Δ^*B*^(*p*, *u*) = Δ^*B*^(*p*) Therefore, the dual indirect function can be expressed as follows:

$$E^{B}\left(p,V(p,y)\right)=\Delta^{B}\left(p\right)E^{A}\left(p,V\left(p,y\right)\right),$$ (2)

where *V* refers to the indirect utility function and *y* refers to total household income.

Eq (2) is equivalent to the following:

$$V^{B}\left(p,y\right)=\frac{V^{A}\left(p,y\right)}{\Delta^{B}\left(p\right)}.$$ (3)

The equivalence scale function is homogenous of degree zero in prices as the expenditure function is homogenous of degree one in prices. Therefore, Blackorby and Donaldson [21] express Eq (3) as follows:

$$V^{B}\left(p,y\right)=V^{A}\;\left(p,\frac{y}{\Delta^{B}\left(p\right)}\right).$$ (4)

Pendakur [22] utilized Roy’s identity to derive the Marshallian demand equations as follows:

$$x_{j}^{B}\left(P,y\right)=x_{j}^{A}\left(p,\frac{y}{\Delta^{B}\left(p\right)}\right)\Delta^{B}\left(p\right)+\frac{y}{\Delta^{B}\left(p\right)}\frac{\partial \Delta^{B}\left(p\right)}{\partial P_{j}},$$ (5)

where $x_{j}^{i}$ refers to the expenditure on commodity group *j* for household *i*.

To obtain the Marshallian expenditure equations, multiply Eq (5) by $\frac{p_{j}}{y}$

$$w_{j}^{B}\left(p,y\right)=w_{j}^{A}\left(p,\frac{y}{\Delta^{B}\left(p\right)}\right)+\eta_{j}^{B}\left(P\right),$$ (6)

where $w_{j}^{i}$ represents the expenditure share of commodity group *j* for household *i*, and $\eta_{j}^{B}\left(P\right)$ is the elasticity of the equivalence scale for household *B* with respect to the price of commodity *j*. As we derived the model using cross-sectional data, the prices are constant and the model can be reduced to the following:

$$w_{j}^{B}\left(y\right)=w_{j}^{A}\left(\frac{y}{\Delta^{B}}\right).$$ (7)

Expressing the commodity group’s expenditure share as a function of household expenditures, including household characteristics and representing the commodity group in Eq (7) yields the following:

$$w_{ij}=f\left(y_{i}-k\delta \right)+z_{i}\beta +\varepsilon_{ij,}$$ (8)

where the function of total expenditure *f*(·) is unknown, *w*_*ij*_ is the log of expenditures on specific good *j* for household *i*, y_i_ is the log of total expenditure, δ is the logarithm of the equivalence scale (Δ), k is the vector of the indicator variables for household composition (i.e., demographics), *β* is a vector of the parameters, *z*_*i*_ refers to other household characteristics (i.e., non-demographic variables), and *E*(*ε*_*ij*_|*z*_*i*_, y_i_) = 0; $var\left(\varepsilon_{ij}|z_{i},y_{i}\right)=\sigma_{j}^{2}$.

### Empirical strategy and estimation methods

To simultaneously examine the impact of total expenditures and the other independent variables, the partial linear model can be used in which the total expenditure function is estimated non-parametrically while linear parametric estimates are calculated for the other variables [23]. However, entering demographics (e.g., household composition) linearly in the partial linear model will impose all expenditure items to take the same Engel curve shape; if one good adopts the shape of the log linear model, the other expenditure categories are restricted to assume the same shape [2], which severely affects the estimated specifications and saturation points across different non-food categories. Therefore, the significance of using equivalized expenditures has emerged, as it permits obtaining flexible shapes across the various expenditure categories.

Maintaining a shape-invariant Engel curve and allowing the demographics to be entered additively into the model have stimulated many studies to adopt the EPLM. EPLM Engel curves exhibit shape invariance across household type [2, 10]. We applied the EPLM and performed two procedures to estimate the parametric part of the EPLM; namely, Robinson’s double residual and Yatchew’s differencing estimators [24, 25].

Following Gozalo [26], Pendakur [22], and Blundell et al. [2], we rescaled expenditures assuming base-independent equivalence scales, which means that the equivalence scale does not differ with the utility level and the Engel curves of a given expenditure share will have identical shapes for different household compositions and can only shift horizontally or vertically [22]. Yatchew [25] proposed a base-independent equivalence scale, *λ*, that is appropriate for describing multiple compositions and providing a shape-invariant curve for a specific commodity. It is suitable for developing countries such as Egypt that have large variations in household size and composition. *λ* takes the following form:

$$\lambda_{}={\left(a+\theta c\right)}^{\sigma },$$ (9)

where *a* is the number of adults, c is the number of children, and *θ* reflects the needs for children relative to adults. It takes into account the fact that weight of a child differs from adults. *σ* measures the economies of scale in expenditure in which any additional member is not counted the same with respect to income needs as household members. We incorporate the demographic composition into the model assuming base independence; we enter the log of the equivalence scale (*λ*) in the partial linear model, and *z* still includes both household composition and other covariates. This yields the following:

$$w_{ij}=f\left({\;y}_{i}-\sigma \;ln(a+\theta c\right))+z_{i}\beta +\varepsilon_{ij}.$$ (10)

To avoid using different equivalent household sizes for each expenditure category, which, in turn, causes difficulty in aggregation over all expenditure categories, we apply Yatchew’s [25] procedure in which the equivalence scale is estimated based on all non-food expenditure categories using a grid searching for the parameters that provide the minimum grand total for the sum of the squared residuals for all six expenditure categories.

Endogeneity usually arises in one of three ways: omitted variables, measurement error, or simultaneity [27]. As we confronted in the last two problems in our study, the potential endogeneity in total expenditures can be remedied using two techniques—the control function (CF) approach in a semi-parametric framework. The CF approach directly tests the exogeneity of total expenditures and, at the same time, provides a remedy for endogeneity [2, 28].

Eventually, based on semi-parametrically estimated values of the equivalence scale, we estimate the Engel curve relationship for the different expenditure categories using parametric regressions to extract the baseline forms for different expenditure categories. We perform the specification test proposed by Hardle and Mammen [29] to determine whether linear or quadratic parametric models should be used to approximate the semi-parametric fits of various expenditure categories. We tested the performance of many popular specifications of Engel curves (i.e., linear, quadratic, semi-log, double log, working function) and found that the double logarithmic model captured the data structure properly and gave better fits than other specifications based on the Akaike Information Criterion (AIC) and Bayesian Information Criterion (BIC). Therefore, double logarithmic models were used for the null hypothesis in the non-parametric specifications tests. To control also for endogeneity in total expenditures in the parametric regressions, we used the CF approach in which two reduced-form residuals were included in the ordinary least squares (OLS) regression. Two reduced-form residuals were obtained by using log-equivalized income and its square as instruments for log-equivalized expenditure and its square [27, 30].

### Double residual estimator

The conventional technique of P. M. Robinson [24] is widely used to estimate the partial linear model [31–34]. Robinson’s technique depends on non-parametrically regressing both *w*_*ij*_ and the independent variable (z_i_) on y_i_. Then, the estimated residuals resulting from these non-parametric regressions are used to estimate the parametric regression.


$$w_{ij}-E\left(w_{ij}|y_{i}\right)=w_{ij}-E\left(z_{i}|y_{i}\right)\beta -f\left(y\right)=\left[z_{i}-E\left(z_{i}|y_{i}\right)\right]\beta +\varepsilon_{ij},$$ (11)

The non-parametric estimators of *E*(*w*_*ij*_|y_i_) and *E*(*z*_*i*_|y_i_) are produced by bivariate non-parametric local linear regression, ${\hat{g}}_{i}^{w}(y_{i})$ and ${\hat{g}}_{i}^{z}(y_{i})$, as follows:

$$w_{ij}-{\hat{g}}_{i}^{w}\left(y_{i}\right)≅\left(z_{i}-{\hat{g}}_{i}^{z}\left(y_{i}\right)\right)\beta +\varepsilon_{ij},$$ (12)

We perform separate non-parametric regressions for each parametric variable, and ${\hat{g}}_{i}^{z}(y)$ is the estimate of the local polynomial regression of each column of *z* on *y*.

OLS is performed to estimate the vector, *β*, which is $\sqrt{n}$ consistent and asymptotically normal.


$${\hat{\beta }}_{}={\left[\left({z_{i}-{\hat{g}}_{i}^{z}(y_{i}))}^{\prime }{(z}_{i}-{\hat{g}}_{i}^{z}\left(y_{i}\right)\right)\right]}^{-1}{{(z}_{i}-{\hat{g}}_{i}^{z}(y_{i}))}^{\prime }(w_{ij}-{\hat{g}}_{i}^{w}(y_{i})),$$ (13)

Robinson’s estimator is often called the “double residual estimator,” as it includes the residuals from the non-parametric regression of *w*_*i*_ and the independent variable *z*_*i*_ on y_i_.

The estimator of ${\hat{f}}_{i}^{\;}\left(y\right)$ is as follows:

$${\hat{f}}_{i}^{}\left(y_{i}\right)={\hat{g}}_{i}^{w}\left(y_{i}\right)-{\hat{\beta }}^{\prime }{\hat{g}}_{i}^{z}\left(y_{i}\right).$$ (14)

To control for endogeneity in the semi-parametric framework, the mimic approach suggested by Holly and Sargan [30] is used wherein the residual obtained from regressing (*y*) on the instrumental variable x is used as the covariate in the partial linear model, assuming that the linear conditional model holds, as follows:

$$y=x\;\tau +\gamma ,\;with\;E\left(\gamma |x\right)=0$$ (15)

$$w_{j}=f_{j}\left(y\right)+\gamma \rho_{j}+\varepsilon_{j},\;with\;E\left(\varepsilon_{j}|y\right)=0$$ (16)

Then, Eq (16) is rewritten as follows:

$${w_{j}}_{}-E\left({w_{j}}_{\;}|y\right)=\left[\gamma -E\left(\gamma |{\;y}_{\;}\right)\right]\rho_{j}+{\varepsilon_{j},}_{}$$ (17)

The estimator of ${\hat{f}}_{i}^{\;}\left(y_{i}\right)$ is given by the following:

$${\hat{f}}_{i}^{\;}\left(y_{i}\right)={\hat{g}}_{i}^{w}\left(y_{i}\right)-{\hat{g}}_{i}^{\gamma }\left(y_{i}\right){\hat{\rho }}_{}^{}.$$ (18)

The residual is predicted non-parametrically, wherein the partially linear model will contain repressors *z* and *γ* instead of *z* and the parameters (*β*, *ρ*) instead of *β*. As $\hat{\tau }$ and $\hat{\rho }$ converge at $\sqrt{n}$, the asymptotic distribution of ${\hat{f}}_{i}^{}\left(y\right)$ follows the distribution of ${\hat{g}}_{i}^{w}\left(y\right)-{\hat{g}}_{i}^{\gamma }\left(y\right){\hat{\rho }}_{}^{}$.

### Differencing estimator

We used the differencing estimator proposed by Yatchew [25] to estimate the parametric part of the partial linear model. Consider the following:

$$w_{i}=f\left(y\right)+z\beta +\varepsilon ,$$ (19)

Assuming that *E*(*ε*|*z*, *y*) = 0 and the $var\left(\varepsilon |z,y\right)=\sigma_{\varepsilon }^{2}$, *y*’s have been rearranged so that *y*_1_ ≤ ⋯ ≤ *y*_*n*_. Assuming that the conditional mean of *z* is a smooth function of *y*; *E*(*z*|*y*) = *g*(*y*), where *ǵ* is bounded and $var\left(z|y\right)=\sigma_{u}^{2}$, *z* can be written as *z* = *g*(*y*) + *u*. Differencing yields the following:

$$\begin{array}{c} w_{i}-w_{i-1}=\left(z_{i}-z_{i-1}\right)\beta +\left(f\left(y_{i}\right)-f\left(y_{i-1}\right)\right)+\varepsilon_{i}-\varepsilon_{i-1}\\ =(g\left(y_{i}\right)-g\left(y_{i-1}\right))\beta +\left(u_{i}-u_{i-1}\right)\beta +(f\left(y_{i}\right)-f\left(y_{i-1}\right)+\varepsilon_{i}-\varepsilon_{i-1}\\ ≅\left(u_{i}-u_{i-1}\right)\beta +\varepsilon_{i}-\varepsilon_{i-1}, \end{array}$$ (20)

Therefore, the direct effect *f*(*y*) of the non-parametric variable *y* and the indirect effect of *g*(*y*) that exists through *z* are removed. Applying the OLS estimator of *β* to the differenced data yields the following:

$${\hat{\beta }}_{diff}=\frac{\sum (y_{i}-y_{i-1})(z_{i}-z_{i-1})}{\sum {\left(z_{i}-z_{i-1}\right)}^{2}},$$ (21)

Finally, the estimated parametric effect will be removed and the conventional non-parametric technique can be applied to estimate the non-parametric part *f*.


$$w_{i}-z_{i}{\hat{\beta }}_{diff}=z_{i}\left(\beta -{\hat{\beta }}_{diff}\right)+f\left(y_{i}\right)+\varepsilon_{i}≅\;f\left(y_{i}\right)+\varepsilon_{i}$$ (22)

After estimating *β*, local polynomial regression is used to analyze *f* wherein the bandwidths are selected using the cross-validation procedure.

To achieve the highest level of efficiency for differencing estimator, we use higher-order differencing. Consider the following. *m* is the order of differencing, and the differencing weights {*d*_0_, *d*_1_, …, *d*_*m*_} satisfy two conditions as follows: ($\sum_{j=0}^{m}d_{j}=0$, which ensures the removal of the non-parametric effect as the *y*’s become closer, and $\sum_{j=0}^{m}d_{j}^{2}=0$, which ensure that the variance of the weighted sum of residuals equals $\sigma_{\varepsilon }^{2}$). *D* is defined as the differencing square matrix of order *m*, which is the weighted sum of the lag matrices.


$$D=d_{0}L_{0}+d_{1}L_{1}^{\prime }+\cdots +d_{m}L_{m}^{\prime }$$ (23)

By rewriting Eq (19) and applying the *D* matrix, *Dw* = *Df*(*y*) + *Dε*, yields the following:

$$d_{0}w_{i}+\cdots +d_{m}w_{i+m}=d_{0}{f(y}_{0})+\cdots +d_{m}{f(y}_{i+m})+d_{0}\varepsilon_{i}+\cdots +d_{m}\varepsilon_{i+m}$$ (24)

We use moving average differencing weights that reduce bias by its symmetry compared to the optimal differencing weights that decline monotonically in only one direction.

### Poverty measurement

Although there are two major approaches to measuring poverty, the absolute poverty approach is more commonly used than the relative poverty approach in Egypt. The absolute poverty line measures the minimum subsistence required to achieve an acceptable life. It measures the monetary values of predefined goods, ignoring the welfare conditions of other households in the same community [35]. There are four methods that address the absolute poverty concept; namely, the food energy intake (FEI), the cost of basic needs (CBN), the consumption insufficiency method (CI), and the budget standard (BS) methods [35].

By adopting the absolute poverty concept, poverty measurement in Egypt is based on building two separate poverty lines for food and non-food items. The Egyptian food poverty line is always measured using the FEI methodology, which defines the minimum calories needed by a given individual to achieve adequate nutrition, while non-food poverty is measured based on the CBN method [36]. The cost of non-food items is calculated as a whole by scaling up the food poverty line (i.e., it measures the consumption of non-food items required based on poor households whose total expenditure equals the food poverty line) [37]. Simplicity is the primary feature of the CBN method. However, disagreement on using poor households’ expenditures to constitute the required expenditure of essential non-food items led to increasing disapproval of the CBN method’s validity in providing consistent estimates for non-food poverty. The critical aspects of the CBN method can be summarized as follows, CBN measures the non-food poverty rate through a subjective norm by focusing on poor households’ consumption, thus it comprises a severe perception of minimum subsistence and ignores essential non-food items for other households. Furthermore, it assumes that the preference structure in society must be equal to the expenditure pattern of the poor, even though the spending priorities of the poor may differ from other households in society. There are contradictory perceptions about which items are worthwhile expenditures between health, education, housing, and other items.

Critical aspects of the CBN method have prompted the adoption of other approaches. Adopting various poverty lines tests the reliability of poverty estimates. Since poverty is not strictly absolute, and society is the best determinant of the required level of consumption, the estimated saturation point of an expenditure item will provide a societal consensus on the adequate expenditure level required to achieve an accepted social life. It defines the economic level of each household relative to other households in the same society and avoids the difficulties of correctly pricing non-food items. In addition, measuring poverty for a particular commodity gives a specific indicator for each aspect of poverty, in contrast to the total non-food poverty rate calculated by the CBN method, which does not clarify the substitution possibilities that exist while satisfying the various basic needs such that the household’s disposable income is not exceeded, which may result in an underestimation of the poverty rate.

All methods are not free from drawbacks; each method has its own advantages and disadvantages. Achieving integration between methods provides a comprehensive picture of all possible means to alleviate poverty in society and achieve greater prosperity. Our non-food poverty estimates can complement the FEI method’s food poverty estimates, changing the nature of poverty measurement into a mixture between absolute and relative poverty.

### Saturation points and estimation procedures

The consumption of essential commodities can be saturated at a finite level of expenditure. The idea is that the Engel curve has a concave shape for a specific interval of total expenditures in which the expenditure on the commodity becomes insensitive to any further increase in income [6, 9]. The point at which the slope equals zero or at which the curve shifts from concave to convex is the saturation point, which can also be used as the deprivation point (see **S1 and S2 Figs in** S1 File). The minimum level of total expenditure required to satisfy a particular commodity can be derived based on the saturation point’s location. Thus, the saturation point simultaneously indicates the required consumption of a particular commodity and the associated total expenditure needed to meet this commodity. According to this property, households whose total expenditure is less than the minimum total expenditure are considered to be deprived households.

To clarify, consider that there is *j* commodity (*j* = 1) consumed by households. The Engel curve for this commodity has a concave shape. y* is the minimum total expenditure required to achieve the saturation point of *j* commodity; behind this point, household expenditures cease to rise in response to increasing total household expenditure (see **S1 Fig in** S1 File). A deprived household in a specific category is defined as a household that is unable to satisfy the saturation level of a commodity group and consumes less than the saturated expenditure (i.e., total expenditure (*y*_*ij*_) is less than the minimum total expenditure required to satisfy the saturation point of this commodity, $y_{j}^{*}$).

For multiple commodities, this approach can be generalized. Consider *j* = 1,2,…,*k*, where *k* is the number of commodities. The required total expenditure varies according to each commodity, so $y_{j}^{*}$ denotes the minimum total expenditure needed to satisfy the saturation level of the *j*th commodity. Since there are multiple commodities, the minimum total expenditure required to satisfy all of these commodities’ saturation levels is the maximum of the required expenditure levels, $y_{j}^{*}s$, for each of *j* commodities (Max ($y_{1}^{*},y_{2}^{*},\ldots ,y_{k}^{*}$)). The maximum total expenditure of the individual $y_{j}^{*}s$ ensures that all commodities are saturated and used to estimate the overall deprivation index. We illustrate in **S3 Fig in** S1 File the case of three commodities where $y_{1}^{*},y_{2}^{*}$ and $y_{3}^{*}$ are the required expenditure levels to saturate the three commodities. At the expenditure level $y_{3}^{*}$, the consumption of the three commodities is saturated.

Aside from estimating the equivalence scale, the semi-parametric technique provides further verification of the Engel curve’s properties. After removing the parametric effects, the inspection of the non-parametric estimates of total expenditures will consistently indicate the Engel relationship between total expenditure and expenditure on a specific commodity *j* and introduce an indirect approach for estimating the saturation point and determine to what extent the saturation points vary across different expenditure categories. Following Kumar et al. [4, 5], Pal [7], and Sitaramam et al. [8], we estimated the saturating Engel curve, which takes the following form:

$$w_{ij}=\frac{vy_{i}}{k+y_{i}}$$

where ***w***_***i***_ is *i*th household expenditure of commodity group *j*, ***y***_***i***_ is *i*th household’s total expenditure. To control for variation in household composition, we use equivalized expenditures to obtain smoother curves. Kumar et al. [4, 5] supposed that the Engel curve is concave to a specific point, and then it becomes convex with parameters *v* and *k*, where *v* is interpreted as the saturation level of commodity consumption and *k* is the level of expenditure at which the necessity is at half the saturation level. The difference between the saturation level and actual consumption expenditure on a specific commodity equals the amount of deprivation. (*v* − *w*_*i*_/*v*) is the proportional shortfall in consumption from the saturation level. The mean proportional shortfall of commodity consumption from its saturation level is equal to the poverty index. For further details, see Kumar et al. [4, 5] and Sitaramam et al. [8].

## Results

**Fig 1:**
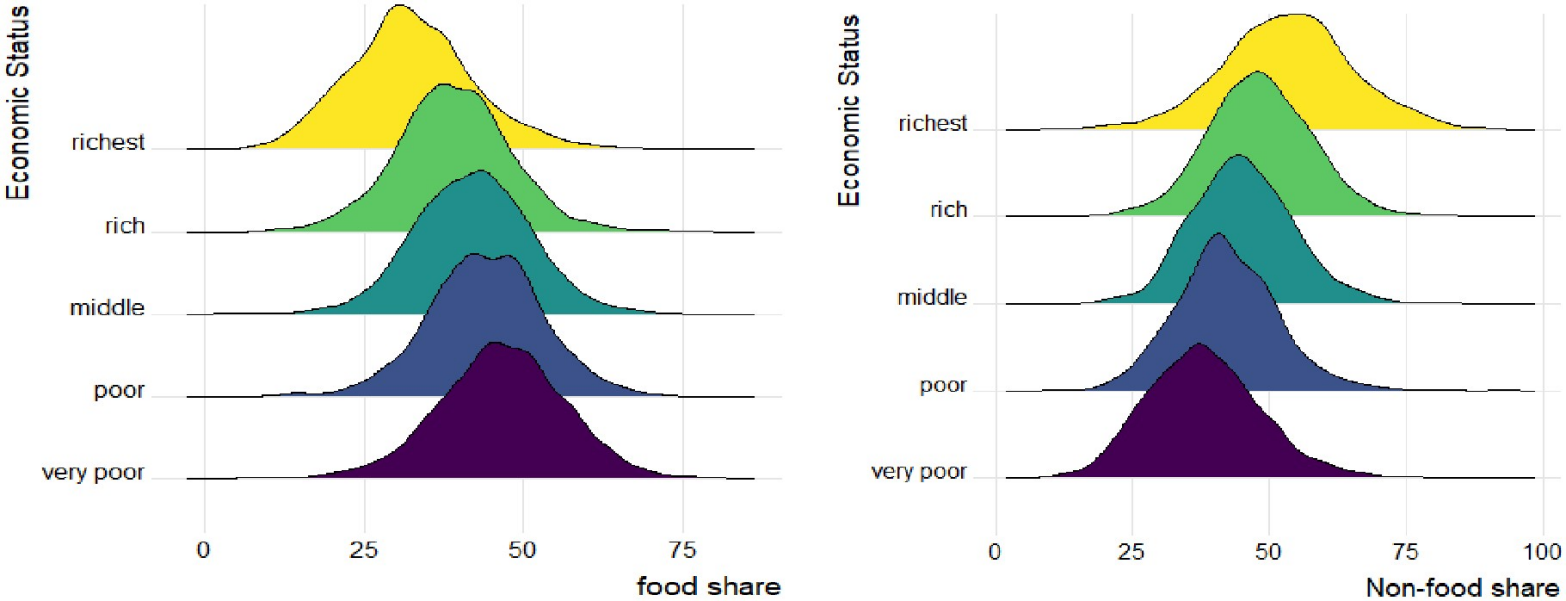
Distribution of food and non-food shares by households’ economic status.

The budget allocation patterns in rural areas reveal that food occupies the first position in expenditure categories; it holds the largest share of total expenditures, accounting for 47.25% and 34.59% of all expenditures for the poorest and the richest rural households, respectively, while the housing category ranks as the next most important item. As shown in Table 3, food share showed a clearly decreasing pattern as the economic status increases in contrast to non-food expenditure shares; education, miscellaneous goods, and services; recreation and culture; and communication, which begin to rise gradually, reach their highest values among the richest households. Fig 1 displays kernel density estimates with optimal bandwidths of food and non-food shares for economically homogeneous households, and it indicates that the non-food share goes up with increasing household economic status, unlike the food share.

**Table 3:** Expenditure shares (%) by economic status.

Expenditure share	Poorest	Poor	Middle class	Rich	Richest
**Food and nonalcoholic beverages**	45.47	44.3	42.5	40.88	35.01
**Alcohol and tobacco**	8.12	8.58	9.09	8.49	7.59
**Clothing and footwear**	4.93	5.62	5.87	6.42	6.86
**Housing and utilities**	17	16.47	15.07	13.33	11.5
**Furniture and housing equipment**	3.67	3.42	3.57	3.72	3.97
**Health**	4.91	5.61	5.79	7.21	10.51
**Transport**	3.34	3.55	4.04	4.67	6.23
**Communication**	1.63	1.54	1.70	1.82	2.04
**Recreation and culture**	0.7	0.86	1.19	1.41	2.55
**Education**	1.75	2.01	2.83	3.53	4.85
**Restaurants and hotels**	3.78	3.38	3.32	3.21	2.92
**Miscellaneous goods and services**	4.67	4.63	4.99	5.26	5.94

Engel stated that there are needs, the satisfaction of which is considered the basis for physical sustenance as nourishment, clothing, housing, heating and lighting, and health, that stand at the top and are called “lower-order needs,” whereas intellectual and spiritual care, legal protection and public safety, and public provisions are second-order needs and called “higher-order needs” [38]. The availability of data for major expenditure groups enables us to investigate whether there is a hierarchy of needs at different economic levels according to Engel’s classification system. We found that the expenditure patterns of low-income households support the importance of physical sustenance that has dominated their consumption patterns. The poorest households allocated large budget shares to food needs (45.47%) according to the well-known “Engel’s law”; followed by housing expenditure share (16.99%) and other first-order needs such health (4.91%) and clothing (4.93%). In the same manner, poor households allocate their budgets, asserting the Engel assumption that low-income households share the same set of needs. Although food and other lower-order needs also account for most of the better-off households’ expenditures, they are able to devote a larger share to higher-order needs such as education (4.85%), transport (6.23%), and recreation and culture (2.55%) than their poor counterparts (1.75%), (3.34%), and (0.7%), respectively.

**Fig 2:**
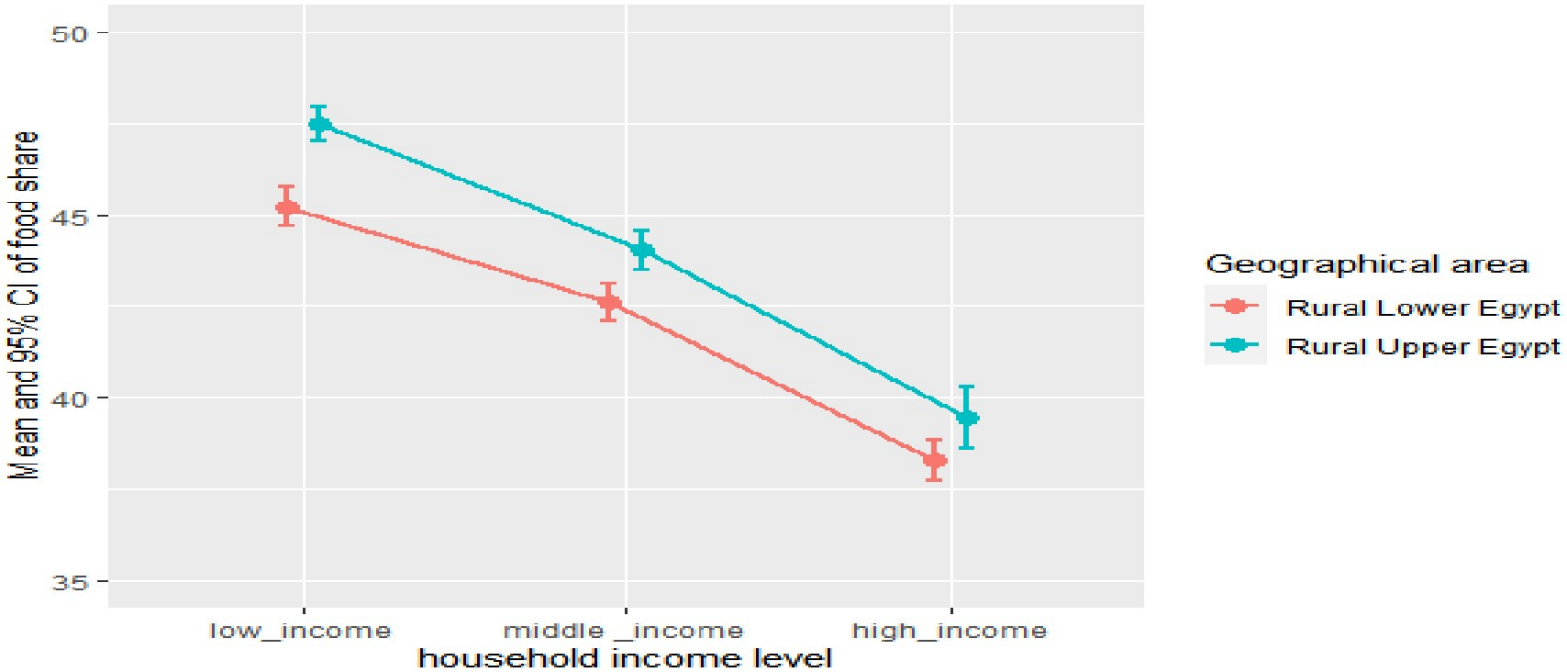
Mean and standard error of food share at different levels of household income by geographical area.

**Fig 3:**
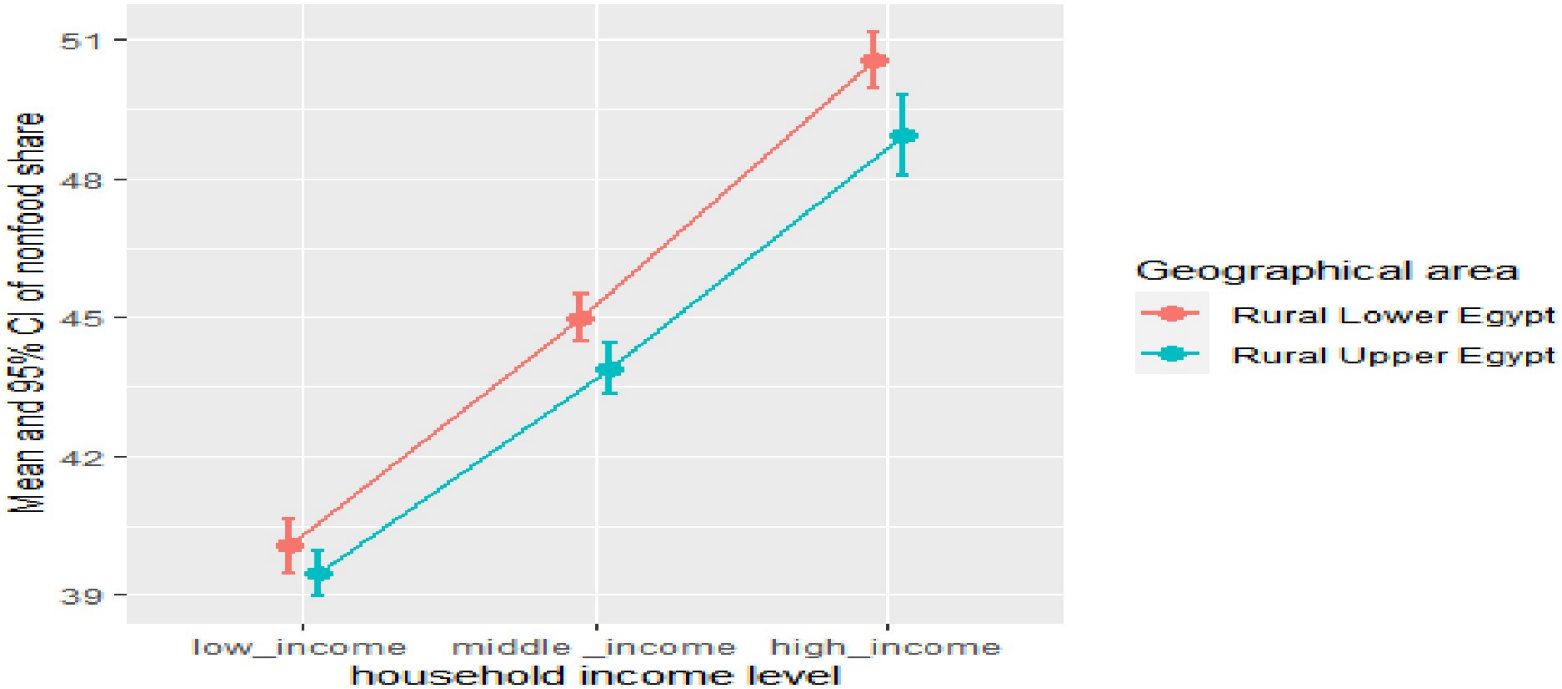
Mean and standard error of non-food share at different levels of household income by geographical area.

**Fig 4:**
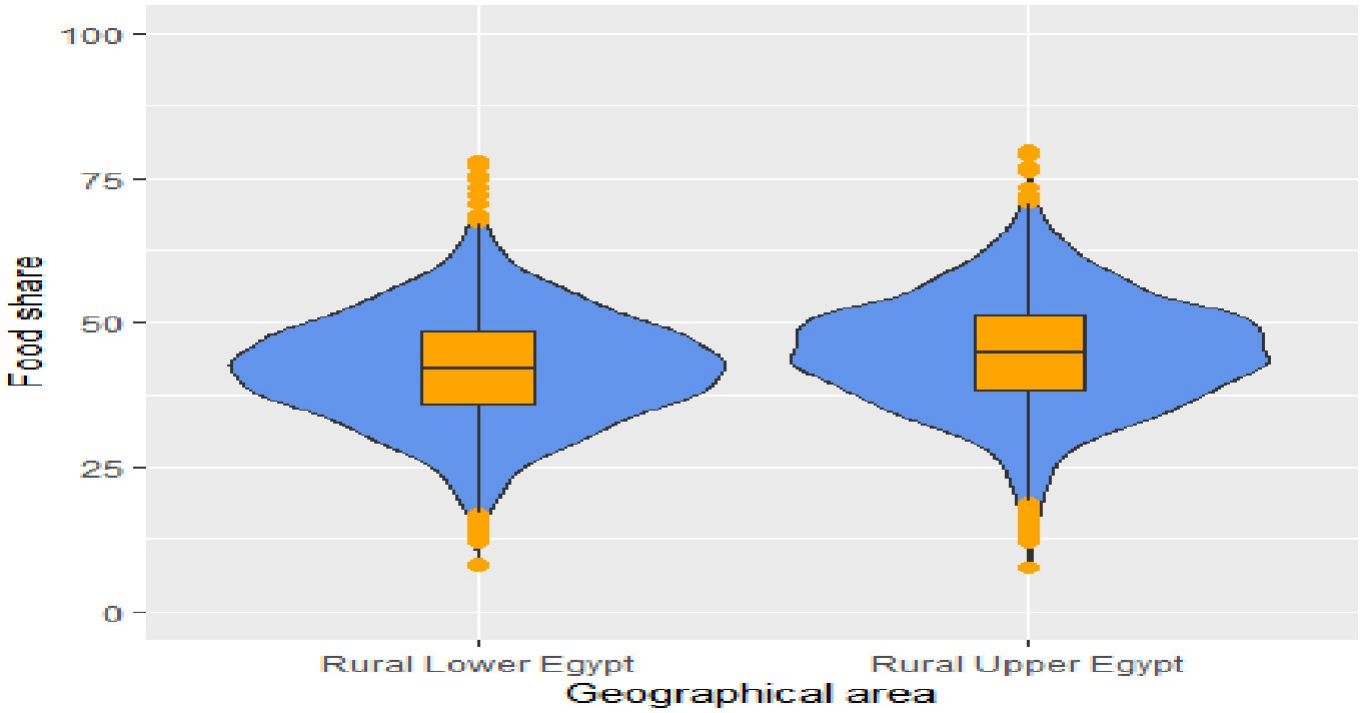
Violin plot with boxplot for food share by geographic area.

**Fig 5:**
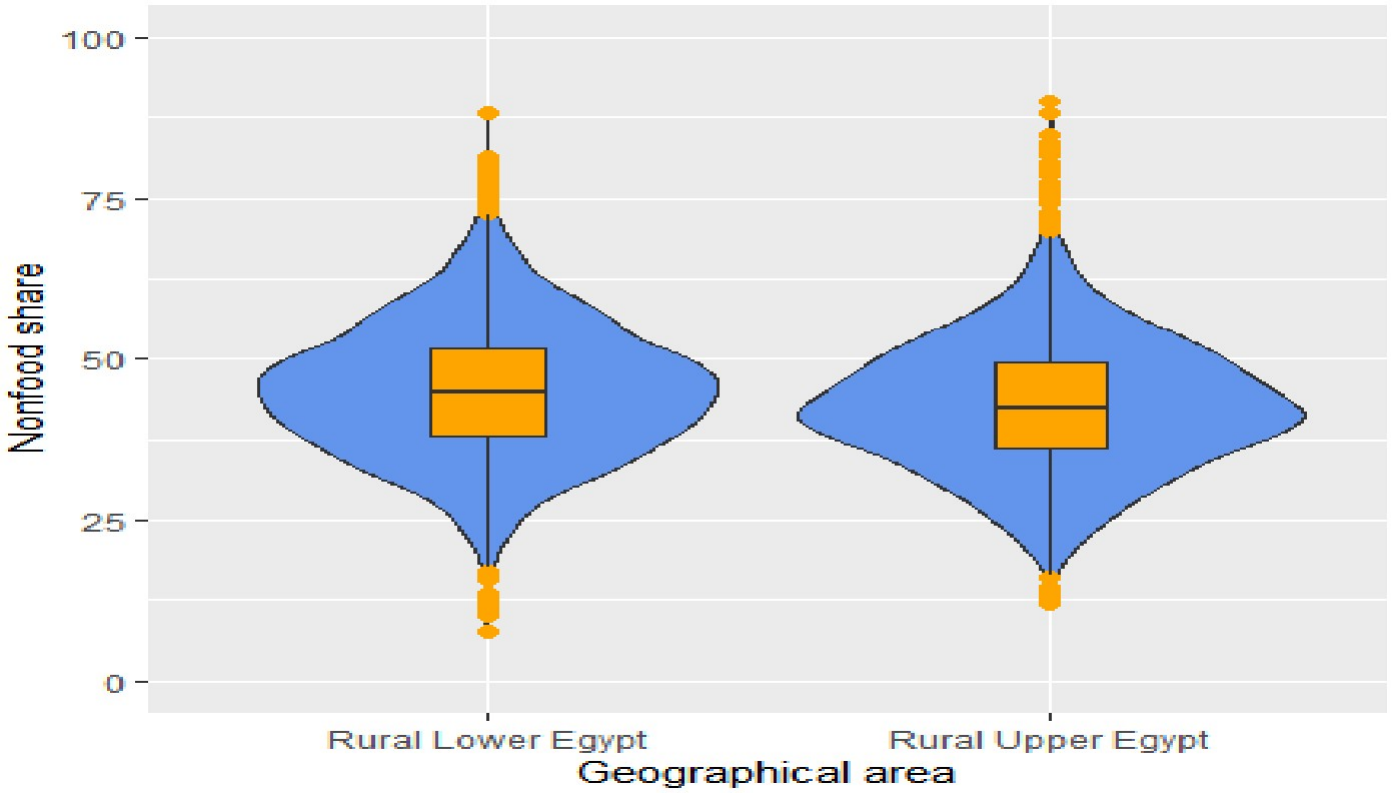
Violin plot with boxplot for nonfood share by geographic area. Violin plots are similar to kernel density plots, but are mirrored and rotated 90°.

**Fig 6:**
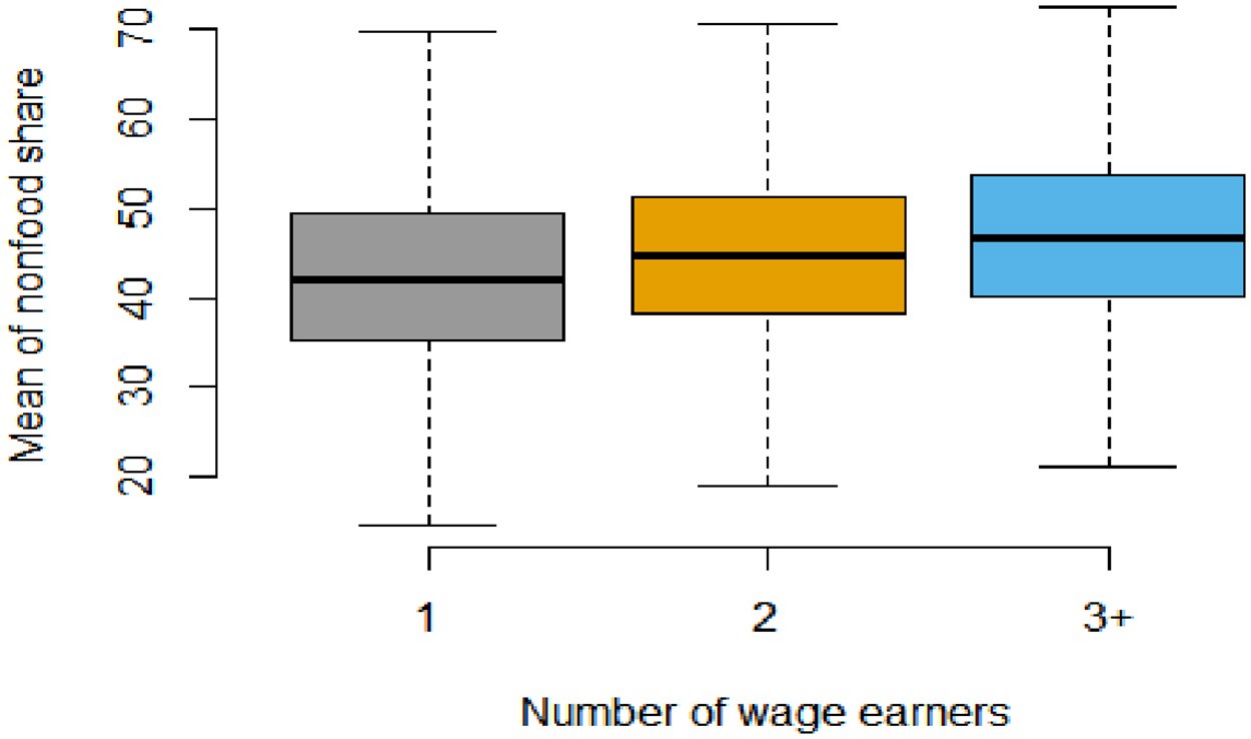
Boxplot of nonfood share by number of wage earners.

Households diversify their spending patterns as their incomes rise. food share decreases while non-food share increase as the household income level increases either in all rural areas, Figs 2 and 3 It is worth mentioning that households in rural Lower Egypt enjoy a relatively better standard of living compared to rural Upper Egypt, and this is evident from their spending pattern that dominated by lower median food share, Figs 4 and 5. As indicated in Fig 6, the higher number of wage earners, the greater the opportunity to spend on non-food needs. Education also exerts a significant effect on the spending pattern in rural areas. Table 4 displays expenditure shares according to the educational level of the household heads. The data detected an inverse relationship between the educational level of the household head and food share; the higher the educational level of the household head, the lower the food share. There is also a significant difference in non-food shares across the educational levels of the household head, the most notable result is that educational expenditure share increases at a rapid rate as the educational level increases. We ascertained that these relationships are not due to a significant association between educational level and income level, but are largely attributable to of the educated head of household’s expenditure priorities. We used a two-way analysis of covariance test to simultaneously evaluate the effect of the two grouping variables (income and educational level) on the various expenditure shares and concluded that both variables are statistically significant (all results are significant at a p-value < .001).

**Table 4:** Distribution of expenditure shares (%) by educational level of household head, rural Egypt.

Expenditure share	Illiterate & read and write	Primary & Lower secondary	Secondary & Post-secondary	University & Postgraduate
Food and nonalcoholic beverages	44.46	41.98	41.71	38.85
Clothing and footwear	4.83	5.75	6.45	7.27
Housing and utilities	17.04	16.50	15.73	15.33
Furniture & Housing equipment	3.65	3.89	4.13	4.29
Health	9.63	8.46	7.38	7.33
Transport	3.65	4.02	4.78	6.24
Communication	1.64	1.78	1.99	2.42
Recreation and culture	1.68	1.69	1.63	2.27
Education	2.75	3.49	4.48	6.08
Restaurants and hotels	3.23	3.13	3.23	3.13
Miscellaneous goods and services	4.88	5.35	5.78	6.42

Table 5 displays the average food and non-food shares across households’ characteristics. Non-food items are considered inevitable needs for even the poorest households, on average, non-food expenditures constitute 37.62% of their budgets versus 47.25% dedicated to food needs. We estimated food and non-food shares individually. As shown in Table 5, food share has a similar response to income changes as the economic status; food share continuously goes down as the income level increases. In contrary to food share, the non-food share has a positive relationship with income and economic status. We found that food and non-food shares differ significantly across income levels, we tested them simultaneously using multivariate analysis of variance (MANOVA), the Pillai test statistic equals 0.985, and a p-value less than 0.001. Non-food share differs significantly across other household characteristics: source of income, number of household earners and household composition. It also differs significantly by head of household characteristics; namely, gender and employment status.

**Table 5:** Mean and standard deviation of food and non-food shares (%) across rural households’ characteristics.

Covariates	Food share^a^	Non-food share^b^
**Economic status (Quintile)**		
Poorest (20%)	47.25 (0.24)	37.62 (0.26)
Poor (20%)	44.71 (0.23)	42.13 (0.22)
Middle (20%)	43.04 (0.24)	44.88 (0.24)
Rich (20%)	40.80 (0.27)	48.17 (0.27)
Richest (20%)	34.59 (0.37)	54.53 (0.41)
**Disposable income**		
Low-income	45.97 (0.19)	40.09 (0.21)
Middle-income	42.91 (0.19)	44.65 (0.20)
High-income	38.43 (0.25)	50.28 (0.27)
**Geographical area**		
Rural Lower Egypt	41.93 (0.17)	45.36 (0.19)
Rural Upper Egypt	44.09 (0.19)	43.21 (0.20)
**Household composition**		
1–2 adults, no children	43.04 (0.37)	40.70 (0.41)
1–2 adults, 1–2 children	42.68 (0.29)	43.66 (0.30)
1–2 adult, 3 or more children	44.28 (0.23)	43.77 (0.24)
3 or more adults, 0-1children	40.75 (0.28)	47.35 (0.37)
3 or more adults, 2–3 children	42.29 (0.34)	47.14 (0.36)
3 or more adults, 4 or more children	46.05 (0.53)	43.98 (0.55)
**Having Member < 14 years**		
Yes	43.53 (0.15)	44.48 (0.16)
No	41.73 (0.23)	44.38 (0.26)
**Having Member > 60 years**		
Yes	44.20 (0.33)	42.45 (0.36)
No	42.57 (0.14)	44.88 (0.15)
**Having ration card**		
Yes	42.99 (0.15)	44.53 (0.16)
No	39.51 (0.82)	46.40 (0.85)
**Source of income**		
Household business	43.49 (0.23)	44.26 (0.25)
Salaries and wages	42.24 (0.17)	45.67 (0.18)
Remittances from country or abroad	43.50 (0.32)	41.68 (0.36)
Other	18.39 (1.65)	27.49 (4.60)
**Number of wage earners**		
1	43.42 (0.17)	43.07 (0.19)
2	42.22 (0.23)	45.72 (0.25)
3+	41.92 (0.34)	47.40 (0.36)
**Age of the household head**		
Young (18–40)	43.95 (0.21)	43.18 (0.21)
Middle aged (40–60)	41.86 (0.19)	46.04 (0.21)
Elderly (over 60)	43.44 (0.31)	42.78 (0.35)
**Gender of household head**		
Male	42.58 (0.14)	45.13 (0.15)
Female	44.27 (0.33)	41.10 (0.37)
**Employment status of household head**		
Employed	42.79 (0.14)	44.96 (0.15)
Unemployed	37.59 (2.52)	49.39 (2.76)
Out of labor force	43.21 (0.29)	42.63 (0.33)
**Household head can read and write**		
No	45.08 (0.22)	42.07 (0.24)
yes	41.73 (0.15)	45.65 (0.17)
**Educational level of household head**		
None	44.46 (0.18)	42.77 (0.19)
Primary & Lower secondary	41.98 (0.33)	45.43 (0.36)
Secondary & Post-secondary	41.71 (0.22)	45.74 (0.25)
University & postgraduate	38.85 (0.48)	48.22 (0.54)

^***a***^: Food share excludes expenses on restaurants (catering services).

^***b***^: Non-food share include expenditure on non-food items and exclude total expenses on durables and actual and imputed rentals for housing so food and non-food shares do not equal 100%.

We performed a comprehensive search for parameters of the equivalence scale; *σ* and *θ* fulfill the minimum grand of the estimated residual variance over the range of 0.1 to 1.0, based on all non-food expenditure categories. The results indicate that the best value of *σ* equals 0.31 and 0.1 for *θ*. Tables 6–8 present the OLS estimates for the six non-food expenditure categories in terms of adult-equivalized expenditures, in addition to estimates of Robinson’s double residual and Yatchew’s differencing methods. Heteroscedasticity-consistent standard errors are also provided. We found that Robinson’s technique has the highest adjusted *R*^2^ while the differencing technique has the lowest *R*^2^ with greater standard errors. The OLS estimates demonstrate that a quadratic fit is significant for the clothing, housing, and transport expenditure categories. The exogeneity of log expenditures across the various expenditure categories is strongly rejected, while the residuals for the square of log expenditures are not significant in almost all of the expenditure categories. Therefore, the reduced-form residual from log expenditure is adequate to control for endogeneity.

**Table 6:** Parametric and semiparametric estimates for housing and clothing expenditures, the Control Function Approach.

	Housing expenditure			Clothing expenditure		
Variable	Parametric estimates	Double residual estimates	Differencing estimates	Parametric estimates	Double residual estimates	Differencing estimates
Log of equivalized total expenditure	5.017*** (0.424)			5.739*** (1.00)		
Squared log of equivalized total expenditure	-0.215** (0.021)			-2.009** (0.050)		
Rural Lower Egypt	0.017 (0.027)	0.018* (0.070)	0.013* (0.083)	-0.223*** (0.019)	-0.319 (0.032)	-0.242 (0.042)
Rural Upper Egypt	0.005 (0.0027)	-0.141* (0.070)	-0.121* (0.091)	-0.037 (0.064)	-0.262* (0.025)	-0.276* (0.012)
Number of Males in the household	-0.028*** (0.004)	-0.000 (0.01)	-0.000 (0.00)	0.057*** (0.010)	0.081** (0.078)	0.092** (0.098)
Number of children in the household	0.002 (0.006)	0.073** (0.028)	0.091** (0.034)	0.052*** (0.023)	0.062* (0.036)	0.089*(0.041)
Number of Elderly in the household	0.006 (0.012)	-0.023 (0.048)	-0.021 (0.057)	-0.025 (0.027)	-0.173 (0.044)	-0.156 (0.087)
Households consist of 1–2 adults, 1–2 children	-0.018 (0.014)	-0.058 (0.061)	-0.052 (0.051)	0.293*** (0.031)	0.440* (0.069)	0.359* (0.075)
Households consist of 1–2 adult, 3 or more children	-0.086*** (0.019)	-0.097 (0.089)	-0.099 (0.093)	0.337*** (0.046)	0.622* (0.034)	0.569* 0.059)
Households consist of 3 or more adults, 2–3 children	-0.105*** (0.016)	-0.092 (0.075)	-0.079 (0.093)	0.205*** (0.038)	0.269* (0.024)	0.301* (0.025)
Households consist of 3 or more adults, 4 or more children	-0.132*** (0.011)	-0.119 (0.038)	-0.120 (0.042)	0.292*** (0.067)	1.396* (0.019)	1.327* (0.028)
Number of earners in the household	-0.059*** (0.006)	-0.019 (0.025)	0.018 (0.036)	0.049*** (0.013)	0.035* (0.093)	0.042* (0.106)
Salaries and wages are the source of household’s income	-0.004 (0.013)	-0.073 (0.057)	-0.098 (0.023)	0.061* (0.031)	0.351 (0.069)	0.332 (0.028)
business is the source of household’s income	-0.0025 (0.014)	-0.012 (0.059)	-0.024 (0.024)	-0.001 (0.032)	0.357 (0.072)	0.327 (0.081)
Age of the HH head	-0.002 (0.002)	0.013 (0.008)	0.019 (0.016)	0.008** (0.004)	0.060 (0.043)	0.078 (0.042)
Square of age of the HH head	0.000(0.00)	-0.00 (0.00)	-0.00 (0.00)	0.000*(0.00)	0.000 (000)	0.000 (000)
Male HH head	-0.029* (0.013)	-0.153**(0.056)	-0.183**(0.043)	-0.147***(0.003)	-0.201***(0.073)	-0.205**(0.075)
HH head has primary education	0.014 (0.011)	0.008 (0.046)	0.006 (0.049)	0.180***(0.026)	0.00324(0.038)	0.00301(0.026)
HH head has secondary education or Post-secondary	0.213**(0.014)	0.187(0.012)	0.189(0.013)	0.002(0.022)	0.082(0.022)	0.087(0.023)
HH head has university or postgraduate education	0.194* (0.028)	0.162(0.069)	0.153(0.026)	0.040*(0.020)	0.030*(0.020)	0.027*(0.035)
HH head is unemployed	-0.021 (0.059)	-0019 (0.070)	-0018 (0.072)	0.068(0.045)	0.053(0.025)	0.021(0.025)
HH head is out of labor	0.033 (0.060)	-0.113 (0.073)	-0.126 (0.089)	-0.036 (0.047)	-0.034 (0.087)	-0.042 (0.090)
Residual	0.322***(0.025)	0.946***(0.054)	0.827***(0.061)	0.565***(0.059)	0.121 (0.086)	0.118 (0.091)
Adjusted *R*^2^	0.38	0.41	0.37	0.49	0.51	0.47

SE(): Heteroskedasticity-consistent standard errors (HCSE) are computed for the semiparametric estimates. HH: household.

**Table 7:** Parametric and semiparametric estimates for health and education expenditures, the Control Function Approach.

	Health expenditure			Education expenditure		
Variable	Parametric estimates	Double residual estimates	Differencing estimates	Parametric estimates	Double residual estimates	Differencing estimates
Log of equivalized total expenditure	2.99* (1.58)			1.128 (0.258)		
Squared log of equivalized total expenditure	-0.093 (0.079)			0.033 (0.116)		
Rural Lower Egypt	1.106***(0.111)	0.961**(0.174)	0.981**(0.189)	0.888 (0.119)	0.261 (0.123)	0.888 (0.121)
Rural Upper Egypt	0.849***(0.121)	0.667 (0.177)	0.721 (0.156)	0.355***(0.122)	0.321***(0.134)	0.355***(0.132)
Number of Males in the household	-0.093***(0.016)	-0.111 (0.076)	-0.123 (0.096)	0.002 (0.018)	0.003* (0.012)	0.002 (0.015)
Number of children in the household	0.023* (0.023)	-0.48*(0.017)	-0.34*(0.022)	-0.136***(0.024)	0.012* (0.014)	-0.136***(0.021)
Number of Elderly in the household	0.176***(0.042)	0.192 (0.082)	0.183 (0.092)	0.0218 (0.065)	-0.038 (0.023)	-0.042 (0.054)
Households consist of 1–2 adults, 1–2 children	-0.200***(0.052)	-0.280 (0.052)	-0.262 (0.021)	0.121*(0.065)	0.110*(0.024)	0.109*(0.065)
Households consist of 1–2 adult, 3 or more children	-0.256***(0.073)	0.229 (0.067)-	0.241 (0.075)	0.673***(0.079)	0.524* (0.071)	0.587* (0.092)
Households consist of 3 or more adults, 2–3 children	-0.035 (0.060)	-0.067 (0.072)	0.079 (0.091)	0.402***(0.067)	0.329* (0.047)	0.398* (0.078)
Households consist of 3 or more adults, 4 or more children	-0.182* (0.106)	-0.052 (0.193)	-0.078 (0.112)	0.591***(0.109)	0.523* (0.114)	0.601* (0.127)
Number of earners in the household	0.0451**(0.021)	0.030 (0.097)	0.026 (0.087)	-0.214***(0.026)	-0.361***(0.078)	-0.281***(0.061)
Salaries and wages are the source of household’s income	-0.275***(0.049)	-0.204 (0.227)	-0.221 (0.186)	0.036 (0.071)	0.047 (0.032)	0.040 (0.047)
business is the source of household’s income	-0.146***(0.051)	0.038 (0.239)	0.042 (0.212)	-0.204***(0.072)	-0.351***(0.022)	-0.301***(0.062)
Age of the HH head	-0.008 (0.008)	0.011 (0.036)	0.016 (0.041)	0.119***(0.011)	0.171***(0.014)	0.159***(0.017)
Square of age of the HH head	0.000*(0.000)	0.000 (0.000)	0.000 (0.000)	-0.001***(0.000)	-0.003***(0.001)	-0.002***(0.000)
Male HH head	0.123*(0.049)	0.368*(0.213)	0.378*(0.262)	-0.199***(0.071)	-0.120***(0.034)	-0.113***(0.066)
HH head has primary education	0.007(0.040)	-0.219(0.053)	-0.219(0.098)	-0.051***(0.047)	-0.098***(0.024)	-0.071***(0.045)
HH head has secondary education or Post- secondary	0.003**(0.002)	0.009**(0.004)	0.01**(0.005)	0.161**(0.014)	0.181**(0.02)	0.179**(0.007)
HH head has university or postgraduate education	0.012(0.004)	0.019*(0.008)	0.017*(0.012)	0.212***(0.012)	0.219***(0.011)	0.209***(0.011)
HH head is unemployed	-0.092(0.219)	-0.190(0.294)	-0.182(0.294)	-0.229 (0.247)	-0.324 (0.214)	-0.287 (0.241)
HH head is out of labor	0.001(0.212)	-0.191(0.293)	-0.194(0.297)	-0.199(0.254)	-0.291(0.210)	-0.271(0.207)
Residual	0.846***(0.092)	0.037***(0.151)	0.032***(0.156)	-0.031*(0.121)	-0.012*(0.098)	-0.017*(0.121)
Adjusted *R*^2^	0.24	0.26	0.24	0.37	0.39	0.38

SE(): Heteroskedasticity-consistent standard errors (HCSE) are computed for the semiparametric estimates. HH: household.

**Table 8:** Parametric and semiparametric estimates for transport and other expenditures, the Control Function Approach.

	Transport expenditure			Other expenditure		
Variable	Parametric estimates	Double residual estimates	Differencing estimates	Parametric estimates	Double residual estimates	Differencing estimates
Log of equivalized total expenditure	3.619**(1.145)			1.144 (0.758)		
Squared log of equivalized total expenditure	-0.263*** (0.057)			0.007 (0.037)		
Rural Upper Egypt	-0.205***(0.074)	-0.238 (0.093)	-0.231 (0.073)	0.036*(0.044)	0.021*(0.027)	0.026*(0.031)
Rural frontier	-0.123* (0.075)	-0.443 (0.091)	-0.424 (0.097)	0.176*(0.045)	0.102*(0.023)	0.114*(0.027)
Number of Males in the household	0.068***(0.019)	0.130** (0.051)	0.141** (0.067)	0.014** (0.006)	0.016** (0.005)	0.016** (0.006)
Number of children in the household	-0.160***(0.017)	-0.058 (0.075)	-0.042 (0.081)	-0.008 (0.008)	-0.005 (0.007)	-0.004 (0.007)
Number of Elderly in the household	-0.055*(0.031)	-0.049 (0.128)	-0.053 (0.119)	-0.061***(0.022)	-0.089***(0.035)	-0.092***(0.034)
1–2 adults, 1–2 children	0.084 (0.038)	0.116 (0.98)	0.121 (0.103)	-0.071***(0.028)	-0.081* (0.022)	-0.089*(0.029)
1–2 adult, 3 or more children	0.101**(0.053)	0.158 (0.043)	0.162 (0.037)	-0.191***(0.026)	-0.091* (0.022)	-0.97*(0.024)
3 or more adults, 2–3 children	0.172***(0.043)	0.074 (0.062)	0.084 (0.056)	-0.078***(0.023)	-0.087* (0.021)	-0.084*(0.031)
3 or more adults, 4 or more children	0.199**(0.078)	0.348 (0.129)	0.327 (0.121)	-0.127***(0.036)	-0.96* (0.022)	-0.103*(0.031)
Number of earners in the household	-0.008(0.016)	0.012 (0.067)	0.023 (0.071)	0.050***(0.008)	0.092***(0.018)	0.088***(0.021)
Salaries and wages	0.273***(0.036)	0.233(0.053)	0.246(0.071)	0.145***(0.026)	0.123***(0.023)	0.125***(0.025)
Remittances from country or abroad	0.0848**(0.037)	0.095 (0.075)	0.071 (0.083)	0.096***(0.024)	0.071***(0.016)	0.079***(0.021)
Age of the HH head	0.022***(0.006)	0.079*** (0.022)	0.057*** (0.027)	-0.004(0.003)	-0.002(0.002)	-0.002(0.001)
Square of age of the HH head	0.000***(0.00)	0.000*** (0.000)	0.000*** (0.000)	0.000(0.000)	0.000(0.000)	0.000(0.000)
Male HH head	-0.077**(0.036)	0.079 (0.049)	0.082 (0.067)	0.079*** (0.023)	0.083*** (0.020)	0.087***(0.032)
Primary education	-0.027 (0.029)	-0.084 (0.54)	-0.091 (0.55)	0.025 (0.015)	0.011 (0.010)	0.014 (0.012)
Secondary education o Post-secondary	0.007*(0.021)	0.098 (0.024)	0.102 (0.029)	0.064(0.012)	0.062 (0.010)	0.061 (0.011)
University or postgraduate education	0.016**(0.041)	0.072*(0.059)	0.076*(0.067)	0.078(0.016)	0.103(0.023)	0.106(0.022)
unemployed	-0.294* (0.160)	-0.406* (0.124)	-0.411* (0.131)	0.078 (0.081)	0.047 (0.032)	0.051 (0.034)
out of labor	-0.302*(0.162)	-0.809**(0.103)	-0.813**(0.107)	0054 (0.084)	0051 (0.082)	0052 (0.082)
Residual	-0.408*** (007)	-0.349***(0.87)	-0.334***(0.75)	-0.027*(0.041)	-0.032*(0.036)	-0.033*(0.035)
Adjusted *R*^2^	0.36	0.38	0.37	0.59	0.61	0.59

SE(): Heteroskedasticity-consistent standard errors (HCSE) are computed for the semiparametric estimates.

**Fig 7:**
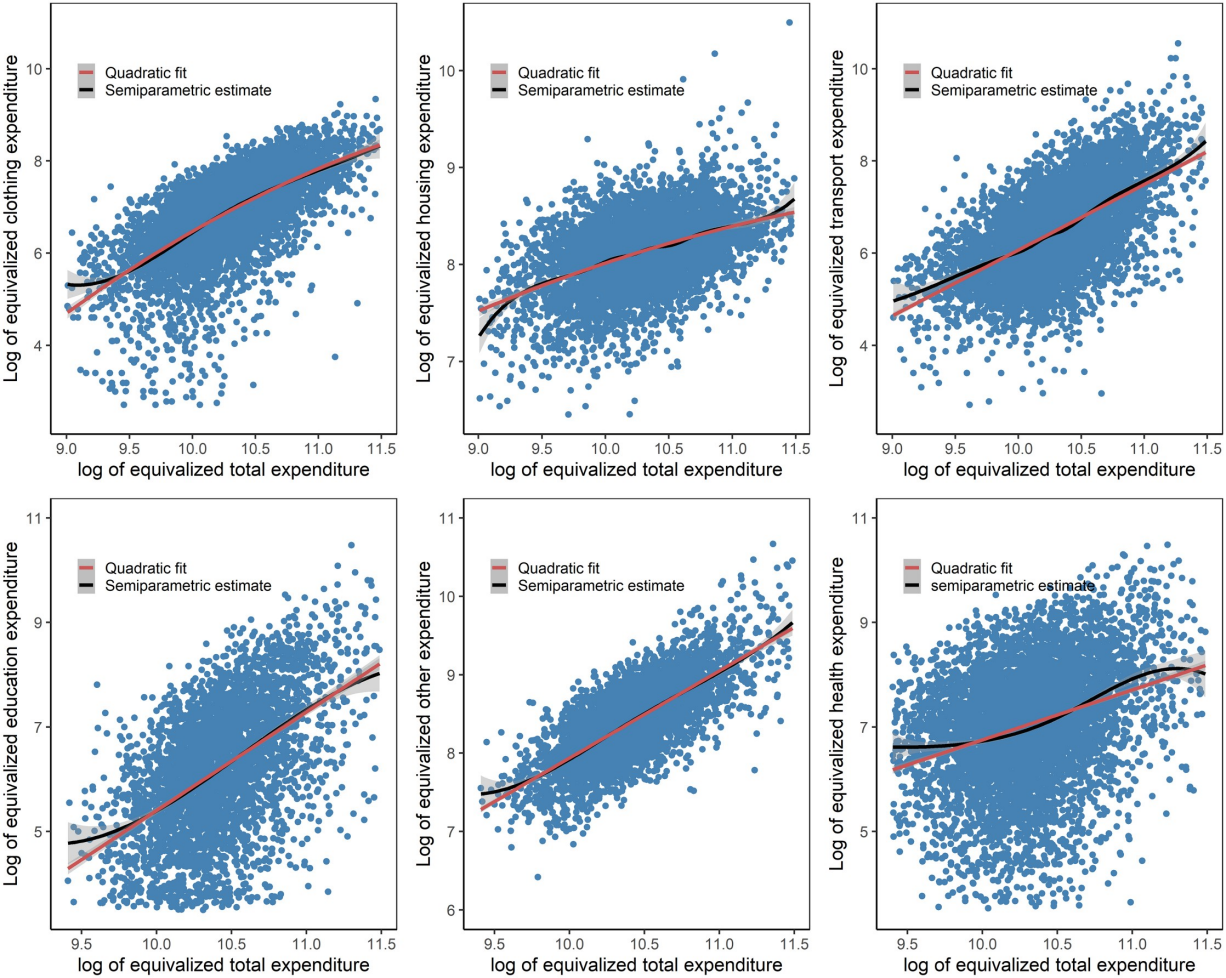
Semi-parametric estimates and quadratic fits for nonfood Engel curves.

Fig 7 shows the non-parametric estimates of total expenditure after removing the parametric effects using Robinson’s technique. It displays the quadratic fit and local polynomial fit with bootstrapped confidence bands for the non-food expenditures groups. We displayed the bootstrapped confidence intervals as they are more accurate than the asymptotic intervals, especially if homoscedasticity has been violated. A graphic comparison of the parametric and semiparametric models reveals the data features. The quadratic fits seem to provide appropriate approximations for almost all of the semiparametric fits of the expenditure categories.

The specification test of Hardle and Mammen [29] also demonstrated that all of the semiparametric estimates of the expenditure categories do not differ significantly from those of the quadratic models. Comparing the semiparametric estimates with the linear fits indicates that the linear fit can provide a reasonable fit for both the transport category and other expenditure categories at the 5% level of significance as shown in Table 9. We also found interesting evidence that assuming the exogeneity of total household expenditure does not have a significant effect on the shape of some expenditure categories (Results available upon request).

**Table 9:** P-value results of Hardle and Mammen tests.

Expenditure category	Linear model	Quadratic model
Clothing	0.05	0.12
Housing	0.00	0.14
Health	0.00	0.18
Education	0.00	0.06
Transport	0.06	0.24
Other	0.09	0.08

**Fig 8:**
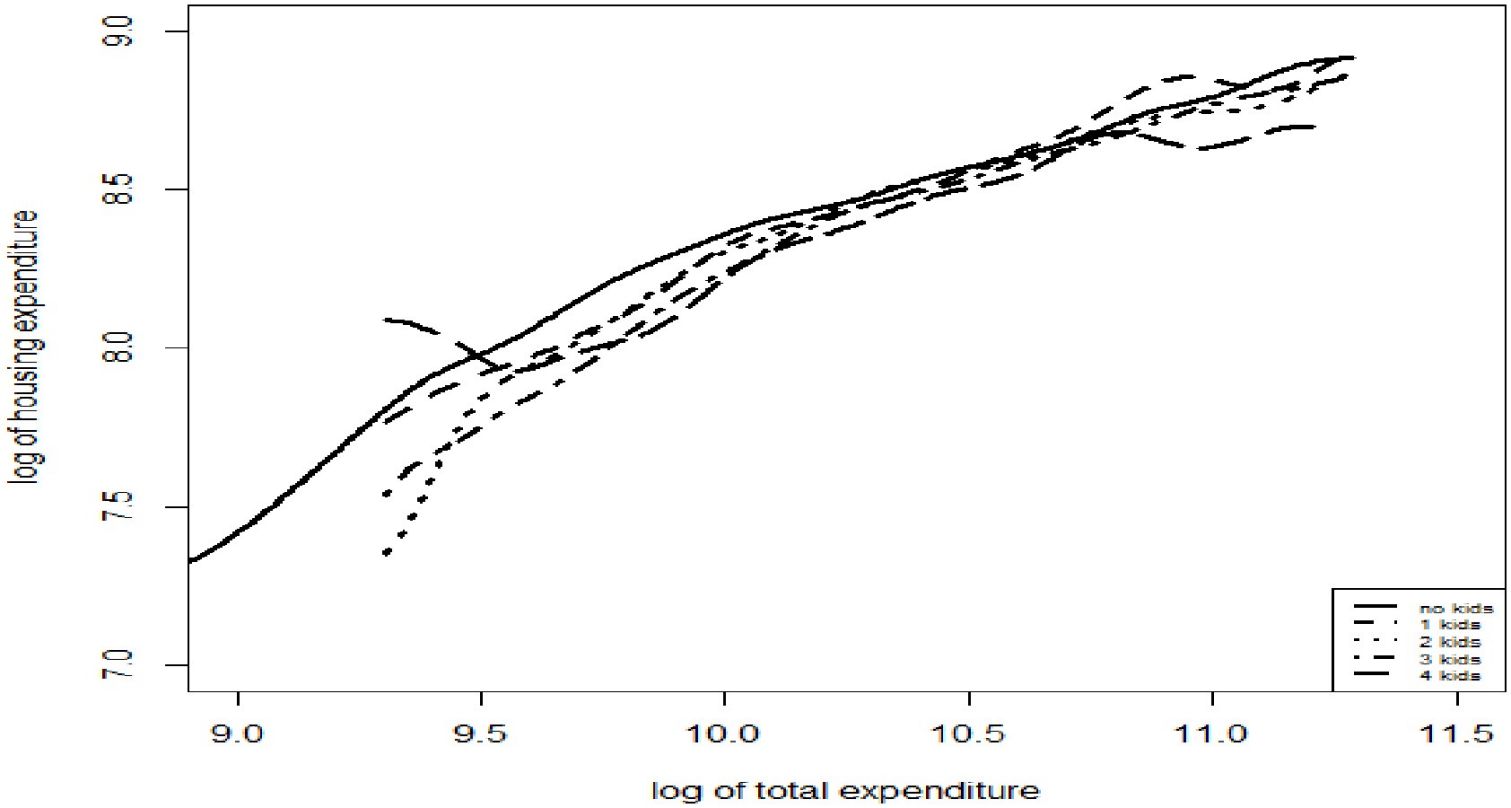
Semi-parametric Engel curves of housing expenditures for households of different number of kids.

It is worth investigating whether the estimated Engel curve for any specific expenditure category is maintained for different demographic groups. Housing expenditure indeed demonstrated that the base-independence assumption holds; it has a similar shape across households with differing numbers of children (see Fig 8). The same results were reached for the other expenditure categories; they show that the base-independence assumption holds for a large segment of the total expenditure distribution.

We truncated the sample and focused on the concave segment following Kumar et al. [4, 5], Pal [7], and Sitaramam et al. [8]. We used an iterative procedure to determine a cutoff point of total expenditures at which the Engel curve transforms from concave to convex using a cubic polynomial between commodity expenditure and total expenditure per adult-equivalent terms. Table 10 provides estimates of the saturation points and the associated minimum total expenditure per adult-equivalent required to satisfy these saturation points. The table also presents the proportion of deprived households and the poverty indices. The initial values of the unknown parameters are derived from the linearized version of the model: *i*/*w*_*i*_ = 1/*v* + (*k*/*v*)(1/*y*_*i*_), which did not yield significantly different estimates than the self-starting function. We found that the estimated saturation functions have lower performance than the semiparametric estimates in terms of the generalized *R*^2^. We also found that the correlation between the predicted values and the actual values did not differ greatly from the values yielded from the double log quadratic models. However, the latter still exhibited a better fit for the data.

**Table 10:** The estimated saturation points for all non-food categories (values are in Egyptian pounds, EGP).

Expenditure category	Saturation point	Minimum total expenditure	Proportion of deprived households	Poverty index	${\overset{-}{R}}^{2}$
Clothing expenditure	1387.68	21128.22	51.4	47.29	41.41
Housing expenditure	4185.06	20312.31	63.51	27.61	37.12
Health expenditure	2210.47	25923,53	63.2	59.48	25.32
Transport expenditure	1903.13	19730.81	58.81	53.21	37.12
Education expenditure	632.02	24921,74	49.74	58.21	34.20
Other expenditure	7821.34	23602,16	68.31	45.7	34.45

* Per adult-equivalent *per annum*, we exclude outliers and badly distorted deprivation estimates. We can strictly compare poverty indices for the different commodity groups as we estimated the equivalent household size based on all non-food commodities.

## Discussion

We found empirical regularity in expenditure on first-order needs at various economic levels but without the same order posited by Engel, where food is the most essential commodity followed by housing, health, and clothing expenditures. A notable decrease in the food share is observed as we move toward higher economic statuses, which is expected. Once a household meets its food needs, it tends to satisfy its non-food needs, especially at higher income levels. While poor households allocate a greater share of their budgets to necessities, rich households allocate a relatively greater share to luxuries.

We investigated the appropriate specifications of the Engel curves for non-food expenditure categories and estimated deprivation indices of non-food needs in rural areas using EPLM, a semiparametric technique that enables non-parametric modeling of the Engel curve relationship, permits the maintenance of shape-invariant Engel curves by controlling household composition, and simultaneously allows non-demographic variables to be entered parametrically. Most of the studies conducted in developing countries have explored the appropriate specification of the food Engel curve [10–13], while specifications for non-food expenditure categories did not receive the same attention. Therefore, our endeavor is among the few studies that have been conducted in developing countries [10]. Our results are in line with Hasan [10], who examined Engel curve specifications for major expenditure categories in Bangladesh in which a quadratic curve provides a reasonable fit for most of the non-food expenditure categories and the ranks of most non-food expenditure categories are of rank three at most.

Our findings summarize the fact that non-food goods are largely consumed by household members at the same time, unlike food goods, which are rivals in consumption. This lower value is highly expected as most non-food goods are public goods that household members can share in consumption, especially in rural areas. The estimated value of *θ* indicates that children’s needs represent only 10% of adult’s non-food needs. Such estimates are rare in developing countries. Our estimates are consistent with other estimates for developing countries. Hasan [10] found that the economy scale, *σ*, equals 0.74 and *θ* equals 0.17. Our estimates are slightly less than his estimates due to the exclusion of the food category in the grid search for both parameters. Moreover, we focus on rural areas, the poorest areas of Egypt, in which the percentage of poor households is 39.7% and even more than half of Rural Upper Egypt (56%).

Although it is widely expected in line with Chai and Moneta [9, 38], Moneta and Chai [6], and Pal [7] that lower-order goods will have the tendency to exhibit saturation in contrast to the higher-order goods and services that have a relatively lower propensity to saturate, the semiparametric fits of the Engel curves for both lower-order categories (clothing, housing, and health) and higher-order categories (education, transport, and other expenditures) have not emerged clearly that they possess saturation; rural households spend more as their incomes increase and both upper and lower bands simultaneously increase. Our findings lie in stark contrast to Pasinetti’s assumption that all types of goods and services will have a saturation level but that the location of the saturation level may vary across commodities and once achieved, household expenditures will not continue to rise in response to an increase in income [39]. This is also contrary to Rao’s [40] hypothesis that the proportion spent on necessities will increase and then become constant, achieving a deprivation point beyond which the expenditure share begins to decrease. However, we found that necessities such as clothing and housing did not tend to saturate at high expenditure levels.

Our results support empirical studies that have shown that strong saturation is largely absent from major expenditure categories except for food expenditure [6]. They elucidated the fact that saturation is lacking due to the different varieties of the same commodity, satisfying the need at higher costs determined by the information and learning process about new consumption techniques [41]. Chai and Moneta [38] similarly indicated that the importance of commodities is no longer the main factor shaping the consumption pattern and resulting in saturation. In light of the fast pace at which product development occurs in modern market economies, there are luxury versions that serve the same need but at higher prices. Therefore, saturating some goods has become captive to the knowledge degree, variable consumer behavior in response to the wide pervasiveness of developed goods [38]. From a contradictory view, we believe that unsaturated curves are largely attributable to the low living standards prevalent in rural areas in which many households live on a subsistence level, so any increase in income is devoted to satisfying other non-food needs.

According to the non-linear least square estimates of the saturating Engel curve, after truncating the sample with a special focus on the concave segment [4, 5, 7, 8], we found that the health and education categories have the highest required total expenditures compared with the other expenditure categories of 25,923 EGP and 24,921 EGP, respectively. While the minimum expenditure level required to satisfy housing necessity (*k*) is 20,312 EGP *per annum* and its saturation point is 4185.06 EGP *per annum*, which implies that only 27.1% of rural households are considered poor in the housing category.

The health category witnessed the highest poverty index. Our results can be attributed to several reasons. First, Egypt invests a small percentage of its budget in the health sector (which does not exceed 5.5% of total government budget), synchronized with a heavy reliance on out-of-pocket health payments. According to National Health Accounts (NHA) in Egypt, out-of-pocket health payments accounted for more than 70% of the health spending in Egypt. These figures are completely opposite from the high-income countries in which the government spending exceeds 70% while out-of-pocket payments are less than 25% of total health spending. Despite the annual decline in health public investment, urban areas have accounted for the largest proportion of public health spending in contrast to rural areas; the spending gap between urban and rural areas reached 67%. Moreover, the HIEC survey revealed a high prevalence of chronic disease in rural areas; every rural household had at least one chronically ill member, and the average number of household members with a chronic disease is three. It also indicted a low level of health insurance coverage; about 68% of household heads are uninsured even though 71.64% of them have at least one chronic disease. This supports the fact that a large proportion of rural households need healthcare services but are forced to delay or forgo seeking healthcare to avoid fees and suffer from salient health poverty.

An interesting finding is that rural households allocate small expenditures on the health and education categories even though they have high health burdens, which requires further research on the main factors responsible for depriving them of these basic needs. We suggest that the low-income level and lack of awareness drove these results more than the availability of educational opportunities and subsidized health services in these areas.

Estimating the deprivation index for each expenditure category is considered more helpful for local organizations and policymakers than the CBN method, which measures the overall poverty rate of the non-food group as a whole. Focusing on the specific expenditure categories that possess high deprivation indices will enhance poverty alleviation tools and clarify the main components of poverty and identify the spending groups that need further specific policies. Commodity-specific deprivation indices propose criteria upon which programs targeted at vulnerable households economically can be designed in specific spending categories such as health and education categories in our study. One important implication of our results that health and education items in rural areas require efficient intervention strategies and welfare-improving programs. We hope that reducing commodity-specific deprivation indices will receive its proper place among policy objectives and complement the main objective of reducing overall poverty.

Our applied measure has some limitations. It is empirically impossible that all commodities will possess a saturation point at a specific point of total expenditure onward due to the budget constraints that limit household expenditures to the sum of all expenditures equals total household expenditures, the “adding up restriction.” In addition, at least one commodity will not exhibit a saturation level, especially for high-income households. Similar to other methods, the concept of minimum subsistence is relaxed in our estimated poverty line as all non-food needs are incorporated into the measurement of poverty, proposing a broad concept of poverty. However, this has merits at the same time, as we did not determine the basic non-food items subjectively and avoided exacerbating disagreements about the adequate set.

## Conclusion

Consumption deprivation provides a realistic measure of poverty in which an acceptable level of expenditure can be estimated based on the prevailing consumption patterns. In this context, we utilized the semiparametric technique to correctly describe the consumption patterns of major non-food expenditure categories and measure the equivalence scale to derive deprivation indices for rural areas. The estimated equivalence scale indicates a small relative need of children with respect to adults (0.1) and a large economy scale in non-food consumption (0.31). The most striking finding that the quadratic Engel curve provides a better approximation of all non-food expenditure categories. Our semiparametric analysis demonstrates that most non-food Engel curves exhibited incremental curves in which non-food expenditures were more sensitive to any additional increases in income without any propensity to saturate. Our results thus provide evidence against the most common hypothesis that saturation is present in expenditures on lower-order goods compared with higher-order goods. Based on the parametric estimates of saturated Engel curves of non-food expenditures categories, we found that the incidence of housing poverty is lower, while poverty incidence in the health and education expenditure categories is the highest, clarifying the importance of designing policies for unsatisfied needs that will ultimately contribute to improving households’ living standards and reducing overall poverty rates in general. In future work, we plan to study the impact of the COVID-19 pandemic on non-food consumption in Egypt [42].

## Supporting information

S1 File (DOCX)
