# Supplementary material for: Estimating equivalence scales and non-food needs in Egypt: Parametric and semiparametric regression modeling
Source: PLoS One. 2021 Aug 20;16(8):e0256017. doi: 10.1371/journal.pone.0256017 (PMC8378757; doi:10.1371/journal.pone.0256017)
Supplement: S1 File — (DOCX) [file pone.0256017.s001.docx]

**Supporting Information**

S1 Fig . A saturating Engel curve

Household expenditure on commodity $j$ ceases to rise in response to increasing total expenditure level, the saturation point—the point where the slope equals zero—can be estimated by using the estimated parameters of the quadratic model.

**S2 Fig.** Engel curve shifted from concave to convex

Household expenditure on commodity $j$ turned from concave to convex, thus iterative procedure can be used to determine a cutoff point in total expenditure at which the Engel curve transforms from concave to convex using a cubic polynomial between expenditure of commodity group and total expenditure per adult-equivalent terms. Kumar et al. [4,5] suggested this procedure to truncate the sample and focus on the concave segment.

**S3 Fig**. Engel curve for three commodities and required expenditure levels

**S1 Table .** Description of household expenditure categories

| **Variable Name** | **Variable Label** | **Content** |
| --- | --- | --- |
| FOODEXP | Food and nonalcoholic beverages | Expenses on food and nonalcoholic beverages  All food expenditures include also own grown products expenses and gifts/in-kind received food. Expenses on take away food and meals taken in restaurants. |
| ALCOEXP | Alcohol and tobacco | Expenses on alcoholic beverages, tobacco, and narcotics |
| APPEXP | Clothing and footwear | Expenses on clothing and footwear which include clothes for men, women, and children—clothing material, tailoring charges, footwear expenditure, school clothes, uniforms and footwear |
| HOUSEXP | Housing and utilities | Expenses on housing (actual and imputed rentals and maintenance and repair of the dwelling), water (water supply and miscellaneous services relating to the dwelling), electricity, gas, and other fuels. Renovation costs as well as expenditure on furniture are excluded, as well as expenditure on mortgage (both the capital and interest part). |
| EQUIPEXP | Furniture & Housing equipment | Expenses on furnishings (furniture and furnishings, carpets and other floor coverings), household equipment (household textiles, household appliances, glassware, tableware and household utensils, tools and equipment for house and garden) and goods and services for routine household maintenance. |
| MEDEXP | Health | Expenses on health, including medical products, therapeutic appliances and equipment, outpatient services, and hospital services. Payments for health insurances are excluded |
| TRANEXP | Transport | Expenses on transport, including purchase of vehicles, operation of personal transport equipment, and transport services |
| COMMEXP | Communication | Expenses on communication, including postal services, telephone and telefax equipment,  and telephone and telefax services |
| CULTEXP | Recreation and  Culture | Expenses on recreation and culture, including audio-visual, photographic and information processing equipment, other major durables for recreation and culture, other recreational items and equipment, gardens and pets, recreational and cultural services, newspapers, books and stationery, package holidays |
| EDUCEXP | Education | Expenses on education, including pre-primary and primary education, secondary education, post-secondary non-tertiary education, tertiary education, and education not definable by level. Ideally, corresponds to Code 10 of the COICOP classification.  It covers educational services only and does not include expenditures on educational materials, such as books, catering and transport services etc. |
| RESTOEXP | Restaurants and hotels | Expenses on restaurants (catering services) and hotels (accommodation services). i.e., include three items  SCHRST: School restaurants expenditures  CATSER: Catering services  ACCOM: refer to Accommodation and hotel expenses |
| MISCEXP | Miscellaneous goods and services | Expenses on miscellaneous goods and services, such as personal care, personal effects n.e.c., social protection, insurance, financial services, other services. |
| TNFOOND | Total expenditures on  non-food and nondurables | Alcoholic and tobacco expenditures are included here  TOTEXP – (FOODEXP + TDUR + THOUS)  TDUR: Total expenses on durables  THOUS: Actual and imputed rentals for housing |
| TOTEXP | Total expenditures | Individual consumption expenditure of household is the sum over the expenditure variables above.  total expenditures = FOODEXP + ALCOEXP + APPEXP + HOUSEXP + EQUIPEXP + MEDEXP + TRANEXP + COMMEXP+ CULTEXP + EDUCEXP + MISCEXP + RESTOEXP |

Source**:** Economic Research Forum. (ERF). Available from: <http://www.erfdataportal.com/index.php/catalog/129/data-dictionary>.

*We consider six exhaustive non-food categories; namely, clothing, housing, education expenses, health, transport, and others. Other expenditure categories include furniture and housing equipment, communication, recreation and culture, restaurants and hotels, and miscellaneous goods and services.
